# Development and validation of an online dynamic nomogram based on the atherogenic index of plasma to screen nonalcoholic fatty liver disease

**DOI:** 10.1186/s12944-023-01808-0

**Published:** 2023-03-29

**Authors:** Hewei Peng, Junchao Zhang, Xianhua Huang, Miao Xu, Jingru Huang, Yunli Wu, Xian-E. Peng

**Affiliations:** 1 Department of Epidemiology and Health Statistics, Fujian Provincial Key Laboratory of Environment Factors and Cancer, School of Public Health, Fujian Medical University, Xuefu North Road 1St, Shangjie Town, Minhou Country, Fuzhou, 350108 Fujian China; 2 Grade 2022, Clinical Medicine Major, Integrated Chinese and Western medicine school, Fujian University of Traditional Chinese Medicine, 350108 Fuzhou, China; 3 Key Laboratory of Ministry of Education for Gastrointestinal Cancer, Fujian Medical University, Fuzhou, 350108 China

**Keywords:** Nonalcoholic fatty liver disease, Dynamic nomogram, Noninvasive models

## Abstract

**Background:**

Nonalcoholic fatty liver disease (NAFLD), a common liver disease worldwide, can be reversed early in life with lifestyle and medical interventions. This study aimed to develop a noninvasive tool to screen NAFLD accurately.

**Methods:**

Risk factors for NAFLD were identified using multivariate logistic regression analysis, and an online NAFLD screening nomogram was developed. The nomogram was compared with reported models (fatty liver index (FLI), atherogenic index of plasma (AIP), and hepatic steatosis index (HSI)). Nomogram performance was evaluated through internal and external validation (National Health and Nutrition Examination Survey (NHANES) database).

**Results:**

The nomogram was developed based on six variables. The diagnostic performance of the present nomogram for NAFLD (area under the receiver operator characteristic curve (AUROC): 0.863, 0.864, and 0.833, respectively) was superior to that of the HSI (AUROC: 0.835, 0.833, and 0.810, respectively) and AIP (AUROC: 0.782, 0.773, and 0.728, respectively) in the training, validation, and NHANES sets. Decision curve analysis and clinical impact curve analysis presented good clinical utility.

**Conclusion:**

This study establishes a new online dynamic nomogram with excellent diagnostic and clinical performance. It has the potential to be a noninvasive and convenient method for screening individuals at high risk for NAFLD.

**Supplementary Information:**

The online version contains supplementary material available at 10.1186/s12944-023-01808-0.

## Introduction

Nonalcoholic fatty liver disease (NAFLD) is defined as the presence of ≥ 25% hepatic steatosis without significant alcohol consumption or any other secondary causes of fatty liver [1]. With a prevalence of approximately 25% worldwide, NAFLD has become the most common chronic liver disease and an enormous increasing health burden [2]. NAFLD may progress to fibrosis, cirrhosis, liver failure, and hepatocellular carcinoma. However, it can be reversed with lifestyle and medical interventions at an early stage [3–5]. Hence, early detection methods for NAFLD remain urgent.

There are many tools for diagnosing NAFLD. As the gold standard for the diagnosis of NAFLD, liver biopsy has serious real-world limitations, including its invasiveness, high cost, and sample-to-sample variability [6]. Although imaging techniques such as ultrasound, magnetic resonance imaging, and computed tomography could be applied to NAFLD diagnosis, these techniques are expensive and require diagnostic imaging physicians, making them unsuitable for screening large populations. Several noninvasive models to predict the likelihood of NAFLD have been developed, but some are based on biomarkers not included in routine physical examinations [7]. The fatty liver index (FLI) and hepatic steatosis index (HSI) are the two most widely used serologic noninvasive methods to predict NAFLD. They perform well in the detection of NAFLD in different populations but are not completely applicable to the Chinese population [8–10].

The atherogenic index of plasma (AIP) can be used in the auxiliary diagnosis of NAFLD in obese and nonobese individuals [11, 12]. A previous study developed a nomogram based on biochemical and dietary variables to predict NAFLD risk, but some dietary variables may be difficult to obtain [13]. Therefore, this study aims to develop a simpler and more convenient dynamic nomogram based on AIP to screen NAFLD.

## Methods

### Study population

This study was conducted in the health examination center of the Affiliated Nanping First Hospital, Fujian Medical University, from April 2015 to August 2017 using a cross-sectional study method. The research protocol was approved by the Ethics Committee of Fujian Medical University, in line with the Helsinki Declaration (ethics number 2014096).

**Fig. 1:**
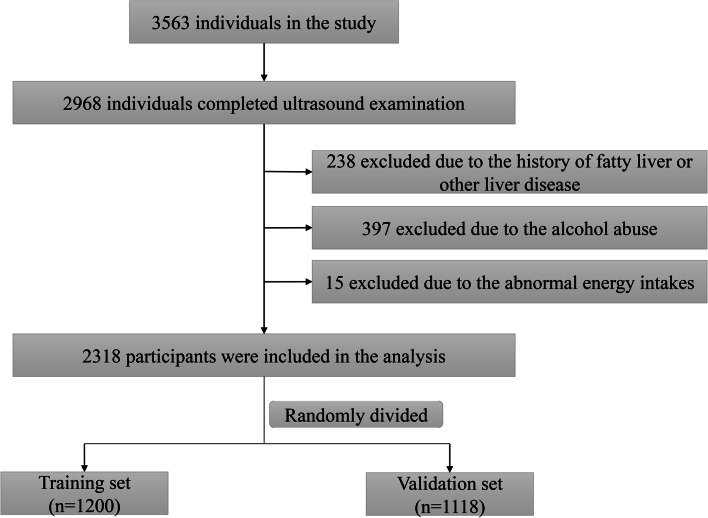
Flowchart of the study participants

The study subjects were permanent residents of Nanping, aged 18 to 74 years, who completed ultrasonography. In addition, exclusion criteria were as follows: (1) Participants who were previously diagnosed with fatty liver or any other liver disease, including viral hepatitis, drug-induced liver disease, autoimmune hepatitis, Wilson's disease, and total parenteral nutrition in the past year. (2) Participants with daily consumption of alcohol > 30 g for men or > 20 g for women [14]. (3) Participants with a daily energy intake of < 600 kcal or > 4,200 kcal for men or < 500 kcal or > 3,500 kcal for women. All of the above information was obtained through face-to-face interviews with participants. The final analysis included 2318 participants randomly divided into two groups (Fig. 1). All participants provided informed consent before they participated in this study.

The National Health and Nutrition Examination Survey (NHANES) for the 2017-March 2020 cycle was used for external validation. It is a cross-sectional study aimed at assessing the health and nutritional status of adults and children in the United States. The study process of including and excluding individuals is shown in Additional file 1. Finally, 5314 individuals were included in the analysis.

### NAFLD diagnosis

NAFLD was diagnosed with abdominal ultrasound by an experienced radiologist based on established criteria without knowledge of laboratory and clinical data. The diagnostic criteria were as follows: (1) The near-field echogenicity in the liver was diffusively enhanced (stronger than that of the kidney and spleen), with gradual attenuation of far-field echogenicity. (2) The intrahepatic lacuna structure is not clearly shown. (3) Hepatomegaly is mild to moderate, with rounded, blunt edges. (4) Blood flow signals in the liver are reduced or even harder to show, but blood flow distribution is normal. If the patients met criterion 1 and one or more of criteria 2–4, the diagnosis of fatty liver was established [15]. In the NHANES population, a controlled attenuation parameter score ≥ 263 dB/m was diagnosed as NAFLD [16].

### Body measurements and laboratory parameters

Anthropometric measurements such as waist circumference (WC), hip circumference (HC), height, and weight were recorded for all subjects. Overweight was defined as a body mass index (BMI) of 24 kg/m^2^ or greater, which was calculated as weight/(height)^2^ [17]. Venous blood samples were collected, and laboratory parameters including fasting plasma glucose, γ-glutamyl transferase, alanine transferase (ALT), aspartate aminotransferase (AST), TG, low-density lipoprotein cholesterol, and HDL-C were measured by trained physicians. Abnormal ALT and AST were defined as ALT > 40 IU/L and AST > 40 IU/L, respectively [18]. AIP was calculated by log (TG/HDL-C) and classified by tertiles (low: < -0.017, median: -0.017—0.049, high: > 0.049) [19]. FLI and HSI were calculated using published formulas [8, 9].

### Concomitant disease diagnosis

Blood pressure was measured in the sitting position using standard equipment. Systolic blood pressure of 140 mmHg or greater, diastolic blood pressure of 90 mmHg or greater, or taking anti-hypertensive medications was diagnosed as hypertension. Diabetes was defined as fasting plasma glucose ≥ 7.0 mmol/L or 2-h postprandial glucose ≥ 11.1 mmol/L.

### Statistical analysis

A random number generator was used to randomly divide the participants into a training set and a validation set. Numbers numbered odd are assigned to the training group, and even numbers are assigned to the validation group. Nonnormal continuous and nominal variables were analyzed using nonparametric Kruskal‒Wallis and chi-square tests for demographic and clinical characteristics of subjects. Multivariate logistic regression analysis was performed to identify independent risk factors for NAFLD. Moreover, the final model was selected using forward stepwise variable selection. The nomogram, which allowed us to derive probability estimates for the presence of NAFLD, was developed based on a logistic regression model obtained in the training set. The scores for each variable were added to calculate a total score, whose axis was obtained at the end of the nomogram. The dynamic nomogram was constructed using the "DynNom" package, which can dynamically predict NAFLD risk on the website.

The area under the receiver operating characteristic curve (AUROC) was calculated to evaluate the nomogram, FLI, HSI, and AIP performances. The calibration curve was performed to evaluate model calibration. The clinical utility of the nomogram, FLI, HSI, and AIP was determined and compared by decision curve analysis (DCA). In addition, a clinical impact curve (CIC) analysis was conducted to reveal the value of the nomogram models more intuitively.

R software, version 4.1.1 and SPSS software, version 19.0.0.1 (IBM SPSS, 2010; Chicago, IL, USA) were used for this analysis. Two-tailed *P* values < 0.05 were considered to indicate statistical significance.

## Results

### Baseline characteristics of participants

As shown in Fig. 1, a total of 2,318 individuals were included in the study and randomly divided into a "training set" (n = 1,200) and a "validation set" (n = 1,118). The demographic and clinical characteristics of the training and validation sets are summarized in Table 1. Participants in the two sets have similar characteristics. Of the 1,200 individuals comprising the training set, 46.2% were male, with a median (interquartile range) age of 43 (31–51). In the validation set, the median age was 43 years, and 507 (45.3) were male. The prevalence of NAFLD in the training set and validation set was 22.7% and 23.0%, respectively.

**Table 1:** Baseline characteristics of the participants in the training set and validation set

Characteristics	Training set (*n* = 1,200)	Validation set (*n* = 1,118)	*P* value
**Demographic characteristics**			
Male (n (%))	554 (46.2)	507 (45.3)	0.693
Age (years, M (IQR))	43 (31–51)	43 (31–51)	0.396
BMI (kg/m^2^, M (IQR))	22.31(20.38–24.45)	22.43 (20.58–24.56)	0.322
WC (cm, M (IQR))	80 (74–87)	80 (74–86)	0.822
HC (cm, M (IQR))	94 (90–98)	94 (91–99)	0.089
SBP (mmHg, M (IQR))	118 (108–126)	118 (108–125)	0.439
DBP (mmHg, M (IQR))	78 (70–84)	78 (70–84)	0.626
Smoker (n (%))	202 (16.8)	161 (14.4)	0.157
Tea drinkers (n (%))	634 (52.8)	570 (51.0)	0.625
Hypertension (n (%))	217 (18.1)	190 (17.0)	0.491
Diabetes (n (%))	59 (4.9)	49 (4.4)	0.542
NAFLD (n (%))	272 (22.7)	257 (23.0)	0.854
**Clinical characteristics**			
GGT (U/L, M (IQR))	20 (15–29)	20 (15–30)	0.675
ALT (U/L, M (IQR))	18 (13–26)	18 (13–25)	0.990
AST (U/L, M (IQR))	20 (18–24)	21 (18–24)	0.734
FPG (mmol/L, M (IQR))	5.13 (4.87–5.43)	5.16 (4.92–5.45)	0.227
TC (mmol/L, M (IQR))	4.98 (4.53–5.51)	4.97 (4.56–5.46)	0.696
TG (mmol/L, M (IQR))	1.15 (0.88–1.59)	1.13 (0.88–1.59)	0.431
LDL-C (mmol/L, M (IQR))	3.11 (2.66–3.57)	3.11 (2.69–3.52)	0.991
HDL-C (mmol/L, M (IQR))	1.37 (1.18–1.47)	1.36 (1.17–1.46)	0.278
AIP (M (IQR))	-0.060 (-0.217–0.127)	-0.069 (-0.217–0.115)	0.655

*BMI* body mass index, *WC* waist circumference, *HC* hip circumference, *SBP* systolic blood pressure, *DBP* diastolic blood pressure, *GGT* γ-glutamyl transferase, *ALT* alanine transferase, *AST* aspartate aminotransferase, *FPG* fasting plasma glucose, *TC* total cholesterol, *TG* total triglyceride, *LDL* low-density lipoprotein, *HDL-C* high-density lipoprotein cholesterol, *AIP* atherogenic index of plasma, *NAFLD* nonalcoholic fatty liver disease, *M (IQR)* median (interquartile range)

### Development of the nomogram

In the training set, shown in Table 2, age (OR = 1.02, 95% CI = 1.01–1.04), WC (OR = 1.09, 95% CI = 1.06–1.12), BMI (overweight vs. nonoverweight, OR = 2.26, 95% CI = 1.49–3.41), serum ALT (abnormal vs. normal, OR = 7.66, 95% CI = 3.81–15.42), and AIP (median vs. low, OR = 1.55, 95% CI = 0.88–2.71; high vs. low, OR = 2.98, 95% CI = 2.04–4.35) were positively associated with NAFLD risk. In contrast, serum AST (Abnormal vs. Normal, OR = 0.27, 95% CI = 0.09–0.83) was inversely related to the risk of NAFLD. Similar relationships were observed in the validation and NHANES sets, except for AST (OR = 0.57, 95% CI = 0.20–1.59 and OR = 1.28, 95% CI = 0.81–2.01, respectively).

**Table 2:** Multivariate logistic regression models in different populations

Variables	Training set OR (95% CI)	Validation set OR (95% CI)	NHANES set OR (95% CI)
Age (years)	1.02 (1.01,1.04)	1.02 (1.00,1.04)	1.01 (1.01,1.01)
WC (cm)	1.09 (1.06,1.12)	1.13 (1.10,1.17)	1.07 (1.06,1.08)
BMI (Overweight vs. Nonoverweight)	2.26 (1.49,3.41)	1.53 (1.01,2.31)	1.88 (1.48,2.39)
ALT (Abnormal vs. Normal)	7.66 (3.81,15.42)	2.24 (1.15,4.40)	2.45 (1.81,3.30)
AST (Abnormal vs. Normal)	0.27 (0.09,0.83)	0.57 (0.20,1.59)	1.28 (0.81,2.01)
AIP			
Low (< -0.017)	1 (Reference)	1 (Reference)	1 (Reference)
Median (-0.017–0.049)	1.55 (0.88,2.71)	2.13 (1.21,3.75)	1.70 (1.33,2.19)
High (> 0.049)	2.98 (2.04,4.35)	3.41 (2.33,4.99)	2.86 (2.48,3.29)

*BMI* body mass index, *WC* waist circumference, *ALT* alanine transferase, *AST* aspartate aminotransferase, *AIP* atherogenic index of plasma, *NAFLD* nonalcoholic fatty liver disease, *NHANES* National Health and Nutrition Examination Survey

**Fig. 2:**
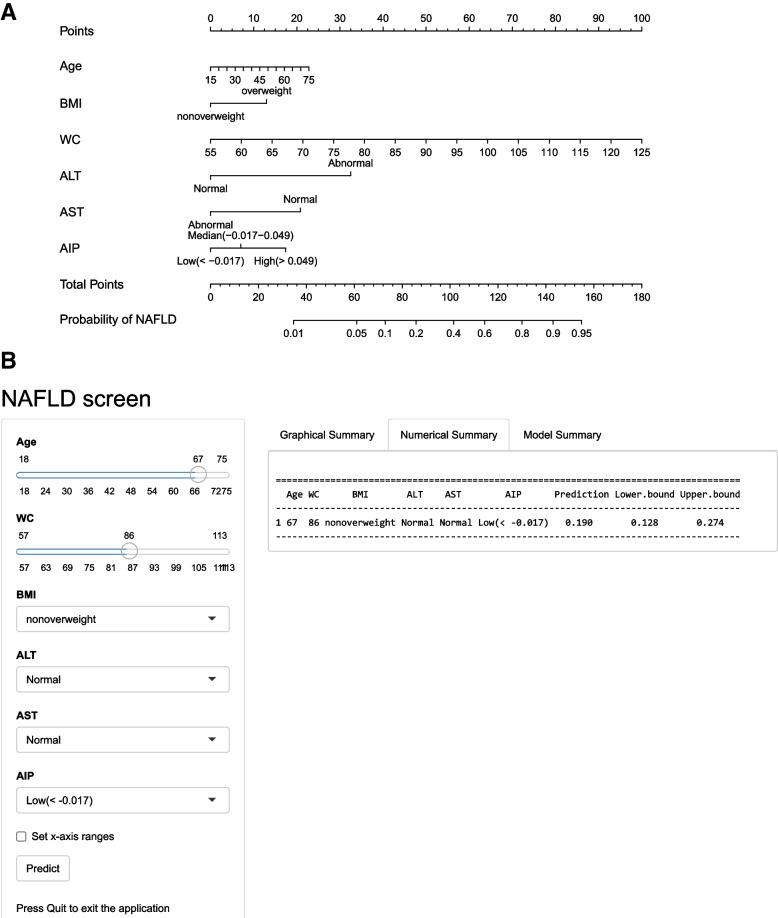
**A** Nomogram developed in the training set for predicting the risk of NAFLD. **B** Online dynamic nomogram accessible at https://fmumodel.shinyapps.io/NAFLD_screen_DN/, depicting an example for predicting the probability of NAFLD for a 67-year-old participant, with BMI = 21.3 kg/m^2^, WC = 86 cm, ALT = 12 IU/L, AST = 20 IU/L, and AIP = -0.07. BMI, body mass index; WC, waist circumference; ALT, alanine transferase; AST, aspartate aminotransferase; AIP, atherogenic index of plasma; NAFLD, nonalcoholic fatty liver disease

As shown in Fig. 2, the final nomogram was developed based on the six variables, including age, BMI (overweight vs. nonoverweight), WC, serum ALT (abnormal vs. normal), AST (abnormal vs. normal), and AIP (low vs. median vs. high), and was available online (https://fmumodel.shinyapps.io/NAFLD_screen_DN/). Each predictor corresponds to a specific score by finding its position on its scale and plotting a straight line to the scale above. The cumulative sum of each "point" is the "total point", which is further converted to the probability of NAFLD. For instance, a 67-year-old participant with a BMI = 21.3 kg/m^2^, WC = 86 cm, ALT = 12 IU/L, AST = 20 IU/L, and AIP = -0.07 had a significant probability of NAFLD of approximately 19.0% (95% CI = 12.8%-27.4%).

### Diagnostic performance of the nomogram

The ROC curves for the nomogram, FLI, HSI, and AIP are shown in Additional file 2. The performance of these models is detailed in Table 3 and Additional file 3. The AUROC of the nomogram in the training set (0.863, 95% CI = 0.840–0.886) was similar to that of the FLI (0.862, 95% CI = 0.838–0.886, *P* = 0.850) and higher than that of the HSI (0.835, 95% CI = 0.808–0.862, *P* = 0.019) and AIP (0.782, 95% CI = 0.752–0.811, *P* < 0.001). Similar significant results were observed in the validation and NHANES sets.

**Table 3:** Diagnostic performance of the nomogram, FLI, HSI, and AIP for predicting NAFLD in the training and validation sets

Models	Training set (*n* = 1200)							Validation set (*n* = 1118)						
	**AUC (95%*CI*)**	***P***	**Youdan**	**Sensitivity**	**Specificity**	**PPV**	**NPV**	**AUC (95% *CI*)**	***P***	**Youdan**	**Sensitivity**	**Specificity**	**PPV**	**NPV**
Nomogram	0.863 (0.840–0.886)	Ref	0.576	0.798	0.778	0.513	0.929	0.864 (0.841–0.887)	Ref	0.583	0.922	0.661	0.448	0.966
FLI	0.862 (0.838–0.886)	0.850	0.564	0.787	0.777	0.508	0.926	0.866 (0.843–0.889)	0.772	0.600	0.851	0.748	0.502	0.944
HSI	0.835 (0.808–0.862)	0.019	0.548	0.812	0.736	0.474	0.931	0.833 (0.806–0.859)	0.006	0.514	0.817	0.697	0.446	0.927
AIP	0.782 (0.752–0.811)	< 0.001	0.444	0.761	0.683	0.413	0.907	0.773 (0.746–0.808)	< 0.001	0.444	0.767	0.677	0.415	0.907

*AUROC* area under the receiver operating characteristics, *PPV* positive predictive value, *NPV* negative predictive value, *NAFLD* nonalcoholic fatty liver disease, *FLI* fatty liver index, *HSI* hepatic steatosis index, *AIP* atherogenic index of plasma, *Ref* reference

Calibration curves indicated great agreement between the probabilities predicted by the nomogram and the actual prevalence of NAFLD in the training set, showing that the nomogram provided good calibration. Good calibration of the model was also confirmed in the validation and NHANES sets (see Additional file 4).

### DCA and CIC for clinical utility of the nomogram

**Fig. 3:**
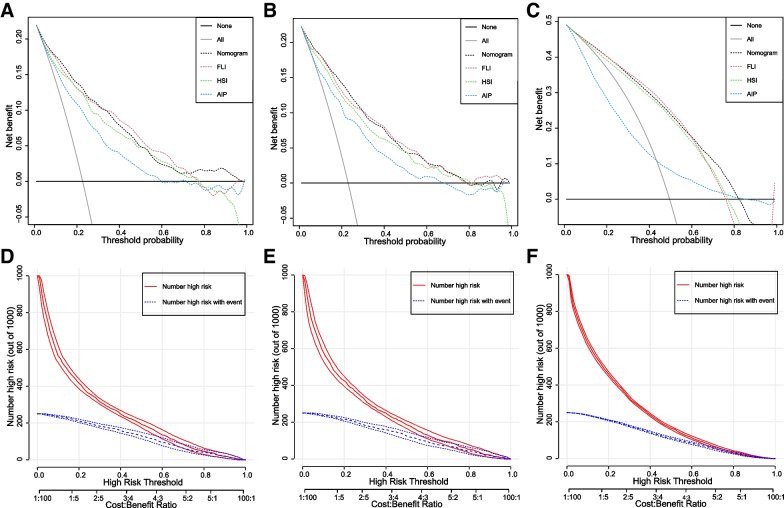
DCA for the nomogram, FLI, HSI, and AIP for prediction of NAFLD, and CIC of the nomogram for prediction of NAFLD. A) DCA in the training set. B) DCA in the validation set. C) DCA in the NHANES set. D) CIC in the training set. E) CIC in the validation set. F) CIC in the NHANES set. DCA, decision curve analysis; CIC, clinical impact curve; FLI, fatty liver index; HSI, hepatic steatosis index; AIP, atherogenic index of plasma; NAFLD, nonalcoholic fatty liver disease

As shown in Fig. 3A, B, and C, DCA was performed to evaluate the clinical relevance of the nomogram in the training, validation, and NHANES sets. In the training set, the nomogram, FLI, HSI, and AIP showed better net benefit than treating all and treating none from a threshold probability of < 100%, < 78%, < 79%, and < 60%, respectively. The nomogram and FLI exhibited the best performance from threshold probabilities of < 33% and > 33%, respectively. In the validation set, from a threshold probability of < 33%, we could obtain more net benefit guided by the nomogram than the referenced strategies (FLI, HSI, and AIP). For example, in the training set, at a threshold of 30%, the nomogram provided a net benefit of 12% (95% CI = 11–14), with a sensitivity of 73% (95% CI = 69–78) and specificity of 82% (95% CI = 80–84), implying that an additional 50% of NAFLD cases could be prevented (standardized net benefit) (see Additional file 5).

The CIC of the nomogram in the training, validation, and NHANES sets (Fig. 3D, E and F) illustrated that the nomogram possesses significant predictive value: the predicted number of high-risk patients was always greater than the number of low-risk patients within the wide and practical ranges of threshold probabilities, and the cost‒benefit ratio would be acceptable in the same range.

### Subgroup analysis

Present model still shows good applicability across strata of age, sex, and presence of diabetes and hypertension (see Additional files 6, 7, and 8). Good calibration and clinical utility of the model were confirmed in participants of different ages (age < 40, 40–60 years, and > 60 years), male, female, diabetes, non-diabetes, hypertension, and non-hypertension groups.

## Discussion

In this study, a novel dynamic online nomogram model based on AIP to screen NAFLD was developed. The final model contained six variables, including age, BMI (overweight vs. nonoverweight), WC, AST (Abnormal vs. Normal), ALT (Abnormal vs. Normal), and AIP (Low vs. Median vs. High). This nomogram model has better performance and clinical utility than the HSI and AIP and is similar to the FLI. The online dynamic nomogram has good diagnostic performance through internal and external validation. The model also shows good applicability in subgroup analysis. Therefore, this dynamic nomogram model may be a valuable screening tool for NAFLD.

NAFLD is one of the most common liver diseases worldwide, and age, obesity, ALT, and AST are important causes underlying NAFLD. In this study, age was positively associated with NAFLD in the training (OR = 1.02, 95% CI = 1.01–1.04), validation (OR = 1.02, 95% CI = 1.00–1.04), and NHANES sets (OR = 1.01, 95% CI = 1.01–1.01). The prevalence of NAFLD increases with age, probably due to the higher susceptibility to oxidative stress and oxidative damage with age [2, 20]. NAFLD is closely associated with obesity, and its prevalence is higher in obese individuals than in nonobese individuals [21, 22]. The present results were consistent with previous studies, showing that BMI was positively associated with NAFLD [23, 24]. Potential mechanisms may be that obesity affects the liver through adipokines (e.g., leptin and adiponectin), hormones derived from adipose tissue. Increased secretion of proinflammatory cytokines and adipokines in visceral adipose tissue and release of free fatty acids into the portal system and systemic circulation lead to dyslipidemia and systemic insulin resistance [22]. ALT and AST are mainly found in liver cells and are often used to indicate the quality of liver function. ALT is closely related to liver fat accumulation and has been reported to be associated with NAFLD [25, 26]. A longitudinal cohort study indicated that the ALT/AST ratio was independently associated with NAFLD in nonobese Chinese people [27].

Several serum-based models have been developed to predict the risk of NAFLD. FLI and HSI are the two most well-established and commonly used indicators of fatty liver [28–30]. FLI was calculated based on triglycerides, BMI, γ-glutamyl transferase, and WC [8]. A study from northern Iran showed that FLI could predict the occurrence of new cases of NAFLD [31]. When validated in the population of the present study, the AUROCs for the training, validation, and NHANES sets were 0.863 (95% CI = 0.840–0.886), 0.864 (95% CI = 0.841–0.887), and 0.833 (95% CI = 0.823–0.844), respectively, which were similar to those of the present nomogram model (*P* = 0.850, *P* = 0.772, and *P* = 0.261, respectively). A meta-analysis was conducted to assess the performance of FLI in detecting NAFLD, and only a weak performance was found [32]. Another commonly used screening tool for NAFLD was the HSI, developed based on Korean populations and calculated using ALT, AST, BMI, sex, and the presence of diabetes mellitus [9]. Nevertheless, the performance of the HSI was significantly lower than that of the nomogram model in the training, validation, and NHANES sets (*P* = 0.019, *P* = 0.006, and *P* < 0.001, respectively). The AIP was positively associated with NAFLD in both obese and nonobese people and was considered to be a new screening indicator of NAFLD [11, 12]. However, the AUROCs of AIP were lower than those of the nomogram model in the training, validation, and NHANES sets (all *P* < 0.001).

Several additional methods were conducted in the present study to evaluate the diagnostic and clinical performance of the nomogram. Calibration curves indicated great agreement between the probabilities predicted by the nomogram and the actual prevalence of NAFLD in the training, validation, and NHANES sets. DCA is a tool for evaluating risk prediction models in a clinical context, and it can assess the utility of models for decision-making [33, 34]. As shown in Fig. 3A and B, in the training set, from threshold probabilities of < 100%, < 78%, < 79%, and < 60%, the nomogram, FLI, HSI, and AIP showed better cost-effectiveness than all-treatment and no-treatment, with nomograms showing the best performance. A similar result was found for a threshold probability < 78% in the validation set. The CIC of the nomogram revealed that the predicted number of high-risk patients was always greater than the number of high-risk patients within the wide and practical ranges of threshold probabilities, and the cost‒benefit ratio would be acceptable in the same range. Subgroup analysis also presented that good calibration and clinical utility of the model were confirmed in participants of different ages (age < 40, 40–60 years, and > 60 years), male, female, diabetes, non-diabetes groups, hypertension, and non- hypertension. These results imply that the present nomogram model has good diagnostic and clinical performance.

### Comparisons with other studies and what does the current work add to the existing knowledge

Compared with other models from previous studies, the indicators used in the current dynamic nomogram are more basic and easier to obtain. The nomogram proposed by the present study is more effective and applicable. It has been verified by internal and external verification that it has good performance and clinical utility.

### Study strengths and limitations

The strengths of this model were as follows. First, the importance of this nomogram is its facility and accuracy in predicting NAFLD. It allows for better visualization of risk prediction than previous ones. The diagnostic performance of the online nomogram was confirmed through internal and external validation. Second, the model is based on a few readily available variables. Hence, it can be applied to the general population or other ethnicities. This model also has limitation that warrant acknowledgment. As liver biopsy is the standard gold method for diagnosing NAFLD, we defined NAFLD using abdominal ultrasonography examination. Recent standardized criteria have significantly improved the diagnostic accuracy of ultrasonography, allowing even mild steatosis to be detected [35]. In addition, in the present study, ultrasound examinations were performed by the same experienced radiologists without knowledge of laboratory and clinical data. Hence, this potential nondifferential bias can only weaken the observed associations. Therefore, this cohort primarily used ultrasonography rather than liver biopsy to diagnose NAFLD.

## Conclusions

In summary, this study developed a novel dynamic online nomogram based on AIP with a relatively excellent predictive ability for screening NAFLD. The nomogram was internally and externally validated and evaluated for its diagnostic and clinical performance by multiple statistical methods. It has the potential to be a noninvasive and convenient method for screening individuals at high risk for NAFLD. Further referral of screened high-risk individuals for other diagnostic tests to confirm NAFLD and thus prevent disease progression through early lifestyle and medical intervention.

## Supplementary Information

**Additional file 1: Figure S1.** Flowchart of the NHANES participants. NHANES, National Health and Nutrition Examination Survey; CAP, controlled attenuation parameter; BMI, body mass index; ALT, alanine transferase; AST, aspartate aminotransferase; TG, total triglyceride; HDL, high-density lipoprotein cholesterol.

**Additional file 2: Figure S2.** Receiver operating characteristic (ROC) curves of the four prediction models. A) Training set. B) Validation set. C) NHANES set. FLI, fatty liver index; HSI, hepatic steatosis index; AIP, atherogenic index of plasma; NHANES, National Health and Nutrition Examination Survey.

**Additional file 3: Table S1.** Diagnostic performance of the nomogram, FLI, HSI, and AIP for predicting NAFLD in the NHANES sets.

**Additional file 4: Figure S3.** Calibration curves of the nomogram for the prediction of NAFLD. A) Training set. B) Validation set. C) NHANES set. NAFLD, nonalcoholic fatty liver disease; NHANES, National Health and Nutrition Examination Survey.

**Additional file 5: Table S2.** Decision curve analysis results of the nomogram at different thresholds.

**Additional file 6: Figure S4.** Calibration curves of the nomogram for prediction of NAFLD in age, sex, diabetes, and hypertension subgroups. A) Age <40 years set. B) Age 40-60 years set. C) Age >60 years set. D) Male set. E) Female set. F) Diabetes set. G) Non-diabetes set. H) Hypertension set. I) Non-hypertension set. NAFLD, nonalcoholic fatty liver disease.

**Additional file 7: Figure S5.** Decision curve analysis of the nomogram for prediction of NAFLD in age, sex, diabetes, and hypertension subgroups. A) Age <40 years set. B) Age 40-60 years set. C) Age >60 years set. D) Male set. E) Female set. F) Diabetes set. G) Non-diabetes set. H) Hypertension set. I) Non-hypertension set. NAFLD, nonalcoholic fatty liver disease.

**Additional file 8: Figure S6.** Clinical impact curve of the nomogram for prediction of NAFLD in age, sex, diabetes, and hypertension subgroups. A) Age <40 years set. B) Age 40-60 years set. C) Age >60 years set. D) Male set. E) Female set. F) Diabetes set. G) Non-diabetes set. H) Hypertension set. I) Non-hypertension set. NAFLD, nonalcoholic fatty liver disease.

## Data Availability

The datasets of the current study are available from the corresponding author upon reasonable request.

## Abbreviations

NAFLD: Nonalcoholic fatty liver disease
NHANES: National Health and Nutrition Examination Survey
FLI: Fatty liver index
HSI: Hepatic steatosis index
AIP: Atherogenic index of plasma
TG: Triglyceride
HDL-C: High-density lipoprotein cholesterol
WC: Waist circumference
HC: Hip circumference
BMI: Body mass index
ALT: Alanine transferase
AST: Aspartate aminotransferase
AUROC: Area under the receiver operating characteristic curve
DCA: Decision curve analysis
CIC: Clinical impact curve
