# Supplementary figures and images for: Development and validation of an online dynamic nomogram based on the atherogenic index of plasma to screen nonalcoholic fatty liver disease
Source: Lipids Health Dis. 2023 Mar 29;22:44. doi: 10.1186/s12944-023-01808-0 (PMC10053077; doi:10.1186/s12944-023-01808-0)

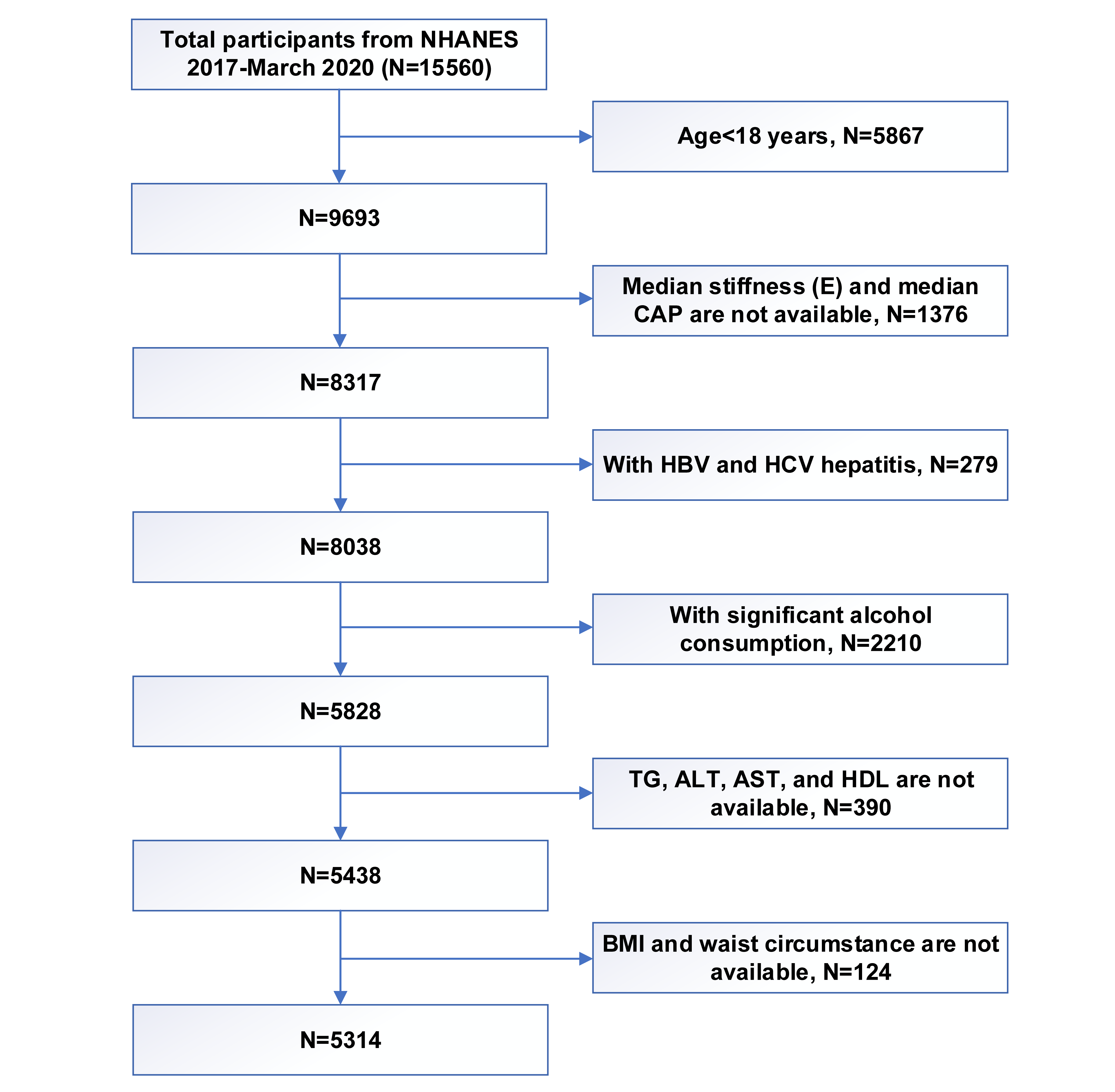

Supplement: Supplementary file 1 — Additional file 1: Figure S1. Flowchart of the NHANES participants. NHANES, National Health and Nutrition Examination Survey; CAP, controlled attenuation parameter; BMI, body mass index; ALT, alanine transferase; AST, aspartate aminotransferase; TG, total triglyceride; HDL, high-density lipoprotein cholesterol. [file 12944_2023_1808_MOESM1_ESM.tif]

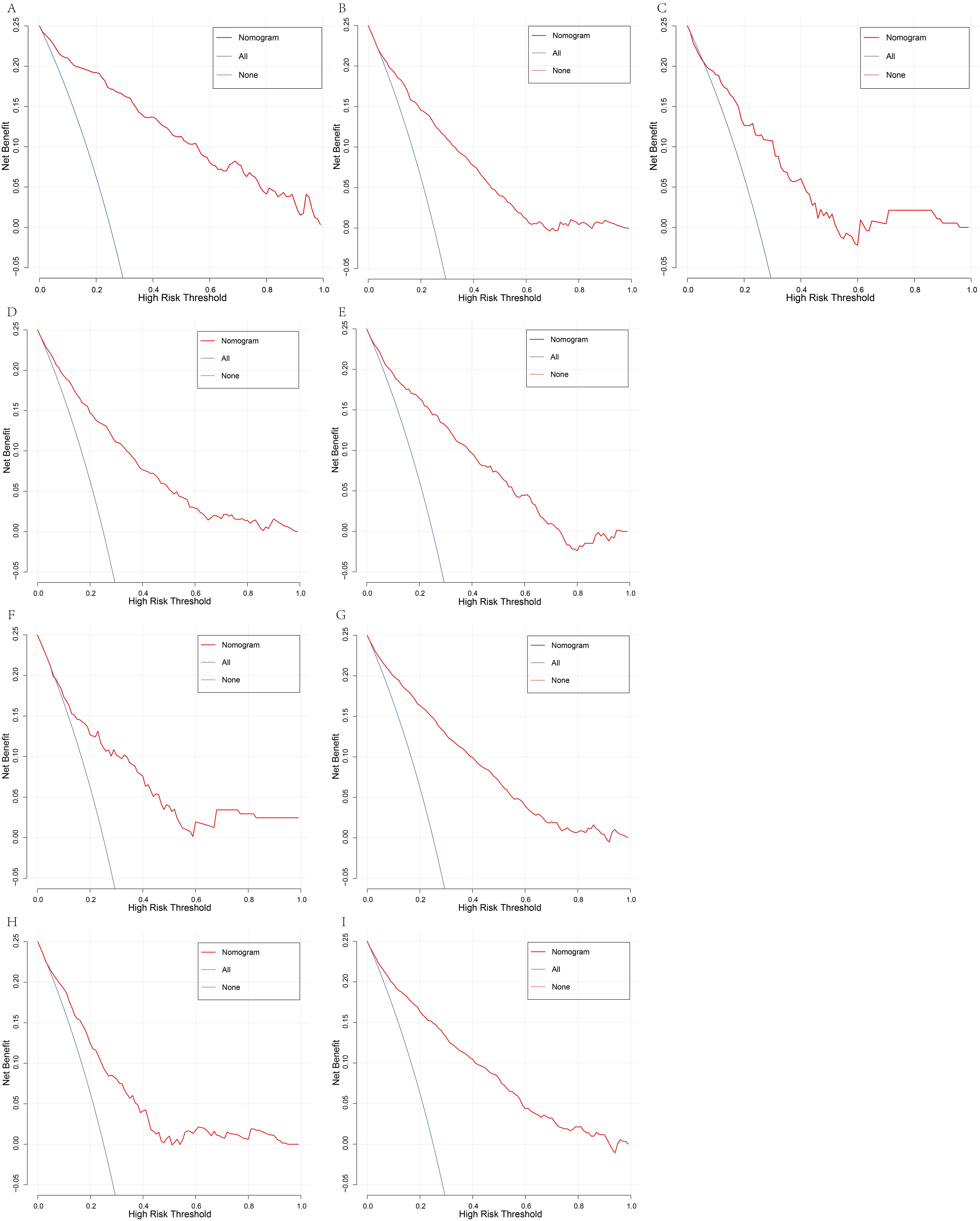

Supplement: Supplementary file 7 — Additional file 7: Figure S5. Decision curve analysis of the nomogram for prediction of NAFLD in age, sex, diabetes, and hypertension subgroups. A) Age <40 years set. B) Age 40-60 years set. C) Age >60 years set. D) Male set. E) Female set. F) Diabetes set. G) Non-diabetes set. H) Hypertension set. I) Non-hypertension set. NAFLD, nonalcoholic fatty liver disease. [file 12944_2023_1808_MOESM7_ESM.png]
