# Supplementary material for: Development and validation of an online dynamic nomogram based on the atherogenic index of plasma to screen nonalcoholic fatty liver disease
Source: Lipids Health Dis. 2023 Mar 29;22:44. doi: 10.1186/s12944-023-01808-0 (PMC10053077; doi:10.1186/s12944-023-01808-0)
Supplement: Supplementary file 3 — Additional file 3: Table S1. Diagnostic performance of the nomogram, FLI, HSI, and AIP for predicting NAFLD in the NHANES sets. [file 12944_2023_1808_MOESM3_ESM.docx]

Table S1. Diagnostic performance of the nomogram, FLI, HSI, and AIP for predicting NAFLD in the NHANES sets

| **Models** | **AUC (95%*CI*)** | ***P*** | **Youdan** | **Sensitivity** | **Specificity** | **PPV** | **NPV** |
| --- | --- | --- | --- | --- | --- | --- | --- |
| Nomogram | 0.833 (0.823-0.844) | Ref | 0.502 | 0.724 | 0.778 | 0.762 | 0.742 |
| FLI | 0.836 (0.826-0.847) | 0.261 | 0.514 | 0.825 | 0.689 | 0.723 | 0.800 |
| HSI | 0.810 (0.798-0.821) | <0.001 | 0.477 | 0.790 | 0.687 | 0.712 | 0.769 |
| AIP | 0.728 (0.715-0.742) | <0.001 | 0.359 | 0.686 | 0.673 | 0.673 | 0.686 |

AUROC, area under the receiver operating characteristics; PPV, positive predictive value; NPV, negative predictive value; NAFLD, nonalcoholic fatty liver disease; FLI, fatty liver index; HSI, hepatic steatosis index; AIP, Atherogenic index of plasma; Ref, reference; NHANES, National Health and Nutrition Examination Survey.
