# Supplementary material for: Development and validation of an online dynamic nomogram based on the atherogenic index of plasma to screen nonalcoholic fatty liver disease
Source: Lipids Health Dis. 2023 Mar 29;22:44. doi: 10.1186/s12944-023-01808-0 (PMC10053077; doi:10.1186/s12944-023-01808-0)
Supplement: Supplementary file 5 — Additional file 5: Table S2. Decision curve analysis results of the nomogram at different thresholds. [file 12944_2023_1808_MOESM5_ESM.docx]

Table S1. Decision Curve Analysis Results of the nomogram at Different Thresholds

| **Risk Threshold** | **Sensitivity** | **Specificity** | **Net Benefit** | **Standardized Net Benefit** |
| --- | --- | --- | --- | --- |
| 0.01 | 1.00(1.00-1.00) | 0.01(0.00-0.09) | 0.24(0.24-0.24) | 0.97(0.97-0.97) |
| 0.05 | 0.98(0.96-0.99) | 0.32(0.25-0.42) | 0.22(0.21-0.22) | 0.87(0.85-0.89) |
| 0.1 | 0.94(0.92-0.96) | 0.56(0.49-0.61) | 0.20(0.19-0.21) | 0.79(0.76-0.83) |
| 0.15 | 0.90(0.87-0.93) | 0.66(0.62-0.70) | 0.18(0.17-0.19) | 0.72(0.68-0.76) |
| 0.2 | 0.84(0.81-0.88) | 0.73(0.70-0.77) | 0.16(0.15-0.17) | 0.64(0.60-0.69) |
| 0.25 | 0.79(0.75-0.83) | 0.78(0.75-0.81) | 0.14(0.13-0.16) | 0.57(0.52-0.63) |
| 0.3 | 0.73(0.69-0.78) | 0.82(0.80-0.84) | 0.12(0.11-0.14) | 0.50(0.45-0.57) |
| 0.35 | 0.69(0.64-0.74) | 0.85(0.83-0.87) | 0.11(0.10-0.13) | 0.45(0.38-0.52) |
| 0.4 | 0.63(0.57-0.70) | 0.88(0.86-0.89) | 0.10(0.08-0.12) | 0.40(0.31-0.46) |
| 0.45 | 0.58(0.52-0.65) | 0.90(0.88-0.91) | 0.08(0.06-0.10) | 0.33(0.25-0.41) |
| 0.5 | 0.52(0.45-0.60) | 0.92(0.90-0.93) | 0.07(0.05-0.09) | 0.27(0.19-0.35) |
| 0.55 | 0.46(0.38-0.54) | 0.93(0.92-0.94) | 0.05(0.03-0.07) | 0.21(0.12-0.30) |
| 0.6 | 0.38(0.31-0.48) | 0.95(0.93-0.96) | 0.03(0.02-0.06) | 0.13(0.06-0.25) |
| 0.65 | 0.32(0.24-0.40) | 0.96(0.95-0.97) | 0.02(0.01-0.05) | 0.10(0.02-0.19) |
| 0.7 | 0.25(0.19-0.34) | 0.97(0.96-0.98) | 0.01(0.00-0.04) | 0.05(-0.01-0.17) |
| 0.75 | 0.20(0.15-0.29) | 0.98(0.97-0.99) | 0.01(0.00-0.04) | 0.05(-0.03-0.15) |
| 0.8 | 0.16(0.10-0.22) | 0.99(0.98-1.00) | 0.02(0.00-0.04) | 0.08(-0.02-0.16) |
| 0.85 | 0.13(0.07-0.18) | 1.00(0.99-1.00) | 0.02(0.00-0.04) | 0.07(0.00-0.15) |
| 0.9 | 0.08(0.04-0.14) | 1.00(1.00-1.00) | 0.02(0.00-0.03) | 0.08(0.00-0.13) |
| 0.95 | 0.03(0.00-0.08) | 1.00(1.00-1.00) | 0.01(0.00-0.02) | 0.03(0.00-0.08) |
